# Recent advances in catalytic synthesis of 2,5-furandimethanol from 5-hydroxymethylfurfural and carbohydrates

**DOI:** 10.1186/s40643-023-00676-x

**Published:** 2023-08-19

**Authors:** Ziting Du, Delong Yang, Qingya Cao, Jinhang Dai, Ronghe Yang, Xingxing Gu, Fukun Li

**Affiliations:** 1 College of Environment and Resources, Chongqing Technology and Business University, Chongqing, 400067 China; 2 Engineering Research Center for Waste Oil Recovery Technology and Equipment of Ministry of Education, Chongqing Technology and Business University, Chongqing, 400067 China

**Keywords:** Biomass, Carbohydrate, 2,5-Furandimethanol, Hydrogenation, HMF

## Abstract

5-Hydroxymethylfurfural (HMF) is a versatile platform chemical derived from the dehydration of renewable carbohydrates (typically glucose/fructose-based monosaccharides, oligosaccharides, and polysaccharides). Some useful compounds, such as 2,5-furandimethanol (FDM), 2,5-dimethylfuran (DMF) and 2,5-dimethyltetrahydrofuran (DMTHF), have been synthesized by reduction of HMF. Among these, FDM is a promising diol and can be further converted towards fine chemicals, liquid fuels and polymer materials. In this review, some typical catalytic systems for the synthesis of FDM from both HMF and carbohydrates were summarized. The discussion focused on controlling the reaction networks for the reduction of HMF. The reaction mechanisms and the stability of the catalysts were introduced briefly. Last but not least, the prospects of effective production of FDM were discussed as well.

## Introduction

Over the last century, the rapid economic development relied on the excessive consumption of non-renewable oil, coal and other fossil resources, resulting in serious resource and environment issues. To seek and utilize green and clean resources has become an urgent need. Biomass is the sole renewable and abundant resource containing organic carbon, thereby being regarded as the best replacement of fossil resources to supply chemicals and fuels for human beings sustainably. The utilization of some typical biomass resources, such as cellulose, hemicellulose, lignin, and chitin, has attracted increasing attention (Torres et al. 2020; Chen et al. 2021; Takkellapati et al. 2018; Dai et al. 2020). Among these, cellulose is the most abundant biopolymer consisting numberous glucose monosaccharide units. Glucose can be further isomerized to fructose. The catalytic conversion of cellulose, glucose and fructose to 5-hydroxymethylfurfural (HMF) has been extensively investigated. HMF contains active furan ring, aldehyde group and hydroxymethyl group. As an important platform compound derived from biomass, HMF can be further converted into a number of useful chemicals via hydrogenation, oxidation, and amination, etc. (Hou et al. 2021). The investigation on the synthesis and conversion of HMF is still a hot research topic.

**Scheme 1.**
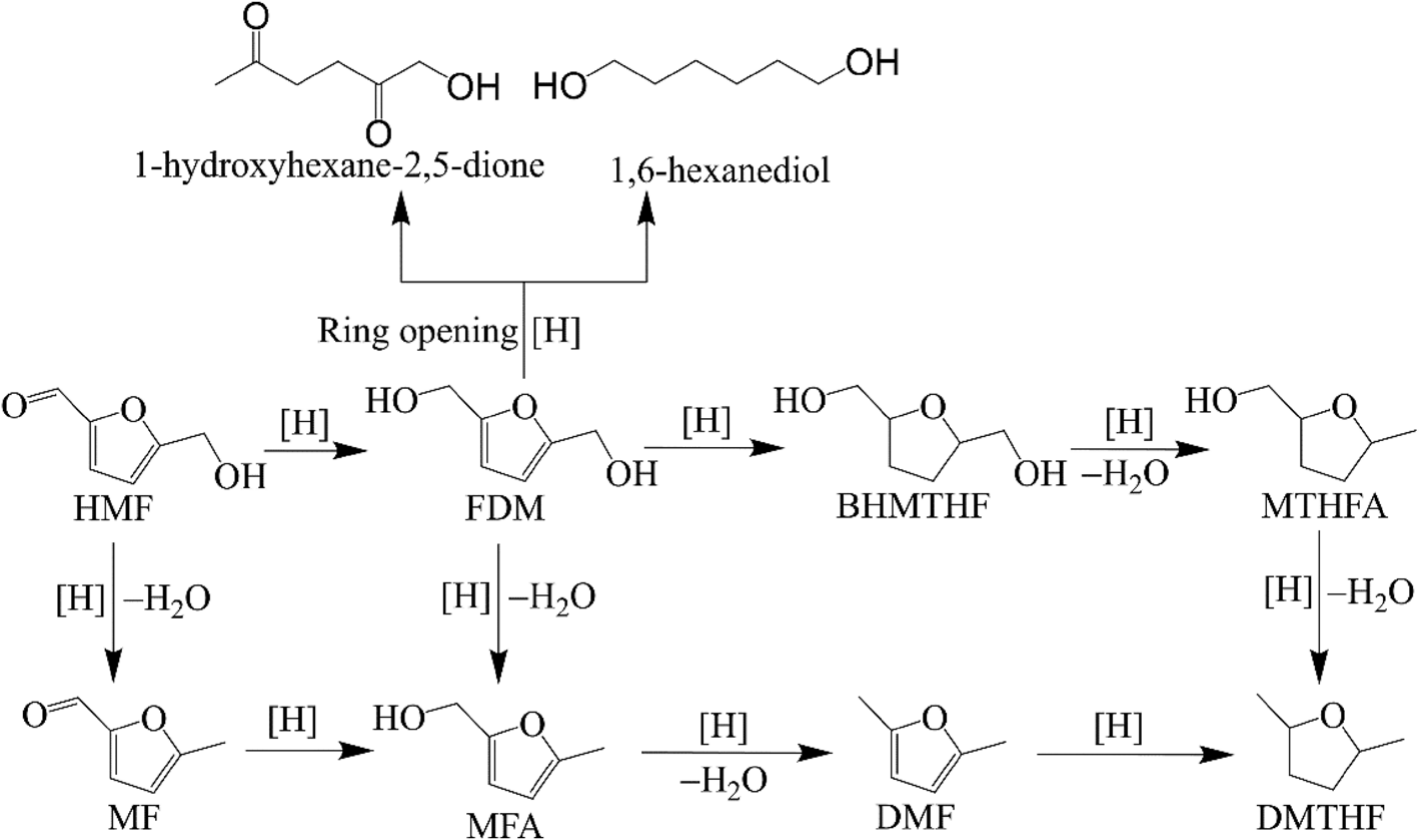
Several reduction products of HMF

The selective reduction of HMF provides several valuable products. For example, 2,5-furandimethanol (FDM), 5-methylfurfural (MF), 5-methylfurfuryl alcohol (MFA) and 2,5-dimethylfuran (DMF) can be obtained by selecive reduction of aldehyde and hydroxymethyl groups with retention of furan ring. Under some conditions, the furan ring of HMF also can be hydrogenated, offering useful products, such as 2,5-bis(hydroxymethyl)tetrahydrofuran (BHMTHF), 5-methyltetrahydrofurfuryl alcohol (MTHFA), and 2,5-dimethyltetrahydrofuran (DMTHF). The ring opening and hydrogenation reactions of HMF yield linear products, such as 1,6-hexanediol and 1-hydroxyhexane-2,5-dione. Some typical reduction products of HMF have been shown in Scheme 1. (He et al. 2022, 2018; Yu 2021; Turkin et al. 2022; Wozniak et al. 2018).

Among these hydrogenated products, FDM is a furan-derivative with two symmetrical hydroxymethyl groups, which has attracted much attention as an important intermediate. The etherification of FDM generates 2,5-bis(alkoxymethyl)furan, a potential biodiesel additive with high energy density and advantageous fuel blending properties (Jae et al. 2014; Lewis et al. 2014; Cao et al. 2014). As a diol, FDM can be used to produce various polyesters (Lillie et al. 2017; Zhang et al. 2017; Jiang et al. 2014). For example, Zhang et al. (Zhang et al. 2017) reported the synthesis of polyesters with high molecular weight using FDM and other bio-based building blocks. The double bonds in FDM unit enhanced the stability of the polyesters. In addition, FDM is a promising synthon for the preparation of biologically active compounds (Gelmini et al. 2016). Previously, there have been some good reviews that summarized the production of FDM from biomass especially HMF (Arico 2021; He et al. 2022; Hu et al. 2018; Tang et al. 2017). However, many novel approaches and exciting achievements for the synthesis of FDM were reported recently. Therefore, a real-time review of this area is still necessary. In this review, we reviewed the recent progress in the synthesis of FDM from biomass-based HMF and carbohydrates and discussed the catalytic paths and catalyst stability.

## The basic properties of FDM

FDM exists as light yellow solid with a density of 1.283 g/cm^3^ at ambient temperature. The melting point, boiling point and flash point of FDM are 74–77 °C, 275 °C and 120 °C, respectively. FDM can be slightly dissolved in acetonitrile and DMSO. Some chemical and physical properties of FDM are listed in Table 1.

**Table 1:** Some chemical and physical properties of FDM

Item	Content
CAS	1883-75-6
EINECS	217-544-1
chemical name	2,5-Furandimethanol
chemical formula	C_6_H_8_O_3_
molecular weight	128.13
InChI	InChI = 1/C_6_H_8_O_3_/c7-3-5-1-2-6(4-8)9-5/h1-2,7-8H,3-4H_2_
density	1.283 g/cm^3^
melting point	74–77 °C
boiling point	275 °C
flash point	120 °C
refractive index	1.542
vapor pressure	0.00248 mmHg at 25 °C
solubility	acetonitrile (slightly), DMSO (slightly)
appearance	light yellow solid

## Synthesis of FDM from HMF

The selective hydrogenation of aldehyde group on HMF towards hydroxymethyl group yields FDM. However, there are several hydrogenation reactions that may occur under true reaction conditions. For example, the aldehyde group, hydroxymethyl group, and furan ring might be hydrogenated, resulting in a mixture of cyclic compounds. In addition, the ring-opening reaction of HMF gives linear molecules. Therefore, it remains a challenge to control the selectivity of hydrogenated products of HMF. Till now, different methods including thermo-catalysis, photo-catalysis, and electro-catalysis have been employed for FDM synthesis from HMF, which will be discussed in this section.

### Synthesis of FDM from HMF by thermo-catalysis

There are two types of hydrogen donors that have been widely used for the conversion of HMF to FDM. First, H_2_ is a typical hydrogen donor with good hydrogenation capacity. Both two hydrogen atoms of the H_2_ molecule can act as hydrogen donors, which meets the requirements of “Green Chemistry”. Moreover, the separation of H_2_ from reaction mixtures can be simply achieved by release of excess H_2_ after hydrogenation reactions. Therefore, H_2_ has been selected as hydrogen donor in biomass conversion in many cases. Nevertheless, some potential safety hazards of flammable and explosive H_2_ drive the development of novel hydrogen resources. Typically, formic acid and some alcohols have replaced H_2_ in some hydrogenation examples, and good yield and selectivity have been obtained. Some of these hydrogen donors (i.e., formic acid, ethanol, etc.) can be produced from renewable biomass-based feedstock, which is attractive. However, the decomposition of these hydrogen donors generates some side-products, resulting in lower atom utilization and difficulties in product separation. Overall, different hydrogen donors have different advantages and disadvantages. The synthesis of FDM from HMF using various hydrogen donors will be discussed next.

#### *H*_*2*_* as reduction reagent*

The conversion of HMF over Ir-, Pd-, Au-, Pt-based catalysts have been reported. Chimentão et al. (Chimentao et al. 2019) studied the selective hydrogenation of HMF over Cl-free Ir/SiO_2_ catalysts at 60 °C for 5 h in tetrahydrofuran (THF) under 10 bar H_2_ pressure, affording 100% selectivity with 60% conversion by inhibiting hydrogenation of the C=C bond. It is notable that chlorine species on Ir/SiO_2_(Cl) catalyst acted as the acid sites to activate the C–O bond, significantly facilitating the hydrogenation to form FDM and enhancing HMF conversion to 97%. The addition of H_2_SO_4_ as a co-catalyst could promote the further conversion of FDM to DMF even DMTHF. The same group (Chimentao et al. 2021) further studied the effect of second metal on Ir/SiO_2_, and the kinetic constants were in the order of Ir–Ni/SiO_2_ > Ir–Co/SiO_2_ > Ir–Ru/SiO_2_ > Ir/SiO_2_, suggesting that the bimetallic catalysts showed higher catalytic activity compared to the monometallic Ir/SiO_2_. The oxophilic nature of the secondary metal might contribute to the higher activity.

The hydrogenation of HMF over Pd-based catalysts also has been demonstrated. There are several important variables that can affect product distribution, such as solvent, co-catalyst, reaction time, and the nature of Pd catalysts. Luijkx et al. (Luijkx et al. 2009) found that the etherification occurred between HMF and alcohols in alcoholic solvents employing 10% Pd/C, and the etherification products acted as intermediates to form DMF. When 1,4-dioxane was used, hydrogenolysis proceeded slowly, and 88.8% selectivity of FDM with 90% conversion was obtained at 60 °C for 740 min. Silva et al. (Silva et al. 2021) used hydrophobic cup-stacked carbon nanotubes (CSCNTs) and hydrophilic micrometric active carbon (AC) to fabricate a hybrid support, and the supported Pd catalysts on this carbonaceous substrate could catalyze the conversion of HMF to FDM with selectivity of $>$ 85%. The electron-donating nature of the support lowered the electron density of Pd element and made reactant adsorption through the C=C double bonds more difficult, thereby increasing the selectivity for hydrogenation of the C=O double bond. In addition, the product selectivity was sensitive to hydrogen pressure, and low hydrogen pressure was required to prevent the over-hydrogenation to obtain high selectivity of FDM.

Pt-based catalysts are efficient for catalytic hydrogenation of HMF to FDM under mild conditions. Chatterjee et al. (Chatterjee et al. 2014) reported the hydrogenation of HMF without any additive over Pt/MCM-41, affording complete conversion with a selectivity of 98.9% in water. The highest selectivity of FDM was obtained under mild conditions (35 °C, 2 h, 8 bar H_2_). The catalyst could be reused at least 7 times without any obvious loss of catalytic activity. The catalytic activity is strongly influenced by the properties of reaction solvents. Among the studied solvents, water is the best choice for the formation of FDM, followed by typical polar protic solvents including methanol, ethanol, butanol and propanol. These polar protic solvents have negative δ values, where the δ value represents the difference between donor number and acceptor number. It was notable that the conversion of HMF decreased with increasing δ value, which might be attributed to the Lewis acidity of these organic solvents. Vikanova et al. (Vikanova et al. 2021) demonstrated that 0.1% Pt/CeO_2_–ZrO_2_ allowed the production of FDM from HMF in 97% yield with 100% selectivity. Ce^3+^ ions seem to be adsorption centers of the carbonyl group, which contributed to high selectivity of FDM. The addition of zirconium species yields defects at the interface, which provides additional sites for the activation of the carbonyl group of HMF. In fact, the acidic and basic sites of the supports played an important role on the hydrogenation of HMF. Wang et al. (Wang et al. 2022a) investigated the effect of different metal oxide supports on FDM synthesis. Interestingly, compared to the typical acidic metal oxide-like TiO_2_, the use of basic metal oxide MgO afforded higher selectivity of FDM (99% vs. 28%), which can be explained by the different types of preferential chemisorption. In brief, the basic sites of metal oxide favored the adsorption of C=O on HMF, which enhanced the selectivity of FDM. Meanwhile, the acidic sites of metal oxide favored the adsorption of C=C group, resulting in poor FDM selectivity. The Pt atoms were responsible for the activation of H_2_. The synergistic effect of basic sites and Pt boosted the formation of FDM. Unfortunately, the used Pt/MgO catalyst showed the decreasing catalytic activity, despite the excellent selectivity of FDM was obtained. The excellent FDM selectivity also could be obtained by confinement effect. For example, Chen et al. (Chen et al. 2020) prepared Pt nanoparticles (NPs) encapsulated into zeolite Y (Pt@Y) as heterogeneous catalysts for the conversion of HMF to FDM, giving nearly 100% yield in water at 80 °C. Good reusability and stability were also obtained. Due to the confinement effect of zeolite Y, the gap in the activation energies between hydrogenation of aldehyde groups and hydrogenolysis of hydroxyl groups was enlarged, and the hindrance effect prevented the adsorption of the furan ring on catalyst surface, resulting in the enhancement of FDM selectivity.

Ohyama et al. (Ohyama et al. 2013) studied the synthesis of FDM from HMF over gold sub–nano-cluster supported on various metal oxides. The acidic sites of metal oxide supports promoted opening furan ring of HMF, leading to the decrease of FDM selectivity. Basic Al_2_O_3_ was found to be favorable for the selective production of FDM. In addition to the acid–base property of supports, Au size is also important. Au sub–nano-clusters were more active than single gold atoms. Over 96% yield of FDM was obtained under 65 bar H_2_ at 120 °C for 2 h.

Some typical examples of selective synthesis of FDM from HMF over noble metal catalysts using H_2_ as donor are summarized in Table 2.

**Table 2:** Some examples of catalytic hydrogenation of HMF to FDM over noble metal catalysts in the presence of H_2_

Catalyst	H_2_ pressure (bar)	Solvent	T (^o^C)	t (h)	Conversion of HMF (%)	FDM yield (mol%)	Refs.
1%Ir/SiO_2_	10	THF	60	5	60	60	(Chimentao et al. 2019)
1%Ir/SiO_2_(Cl)	10	THF	60	5	97	97	(Chimentao et al. 2019)
10%Pd/C	ca.1	1,4-Dioxane	60	12.3	90	79.9	(Luijkx et al. 2009)
2.0%Pd/CSCNT-AC	34	Water	110	2	75	63.8	(Silva et al. 2021)
Pt/MCM-41	8	Water	35	2	100	98.9	(Chatterjee et al. 2014)
0.1% Pt/CeO_2_–ZrO_2_	10	Ethanol	170	2	70	70	(Vikanova et al. 2021)
0.1% Pt/CeO_2_–ZrO_2_	10	Ethanol	170	8	97	97	(Vikanova et al. 2021)
Pt/MgO	20	Isopropanol	120	1	84	83	(Wang et al. 2022a)
Pt/TiO_2_	20	Isopropanol	120	1	28	7.8	(Wang et al. 2022a)
Pt/MgO	20	Isopropanol	150	1	99	98	(Wang et al. 2022a)
Pt@Y	20	Water	80	4	100	100	(Chen et al. 2020)
Au/Al_2_O_3_	65	Water	120	2	–	> 96	(Ohyama et al. 2013)

Some noble metal catalysts have exhibited good catalytic performance for the synthesis of FDM from HMF, but the rareness and high cost of noble metals limit the practical application. Therefore, to develop catalysts using non-noble metal elements is always attractive. Some Co-, Cu-, Ni-, Zr- and Hf-based catalysts have been reported for the reduction of HMF. Arias et al. (Arias et al. 2020) investigated the catalytic performance of monodisperse metallic Co NPs coated with thin carbon shell (Co@C). A 99% selectivity with 97% conversion was obtained in methanol under optimized conditions. The presence of the thin carbon layer suppressed the deep oxidation of Co NPs in air and facilitated their reduction at lower temperature. Compared to polar aprotic solvents, protic solvents (i.e., methanol) favored the polarization of the C=O group, which enhanced the selectivity of FDM. Chandrashekhar et al. (Chandrashekhar et al. 2022) prepared Co–NPs-based catalysts by the immobilization and pyrolysis of Co–terephthalic acid–piperazine metal–organic frameworks on SiO_2_, and the dominant Co element in catalysts existed in metallic form. The as-prepared Co-catalysts were tested for the hydrogenation of HMF to FDM, providing a 95% yield in methanol at 70 °C for 16 h in the presence of 15 bar H_2_. The products could be switched by changing reaction conditions. For example, an 89% yield of MFA was obtained after increasing the temperature and H_2_ pressure to 100 °C and 30 bar, respectively. Wang et al. (Wang et al. 2022c) reported the solvent-free synthesis of FDM from HMF over cobaltic nitrogen-doped carbon (Co–NC) catalysts, and a desirable FDM yield of 91.5% was afforded at 90 °C in the presence of 50 bar H_2_. The doped graphitic N might facilitate the polarization of H_2_ to form H^+^ and H^–^, which promoted the hydrogenation of HMF. This solvent-free system avoids the removal of solvent after reaction. Similar to HMF, furfural also could undergo hydrogenation to form furfuryl alcohol in satisfactory yield employing Co–NC catalysts.

**Scheme 2.**
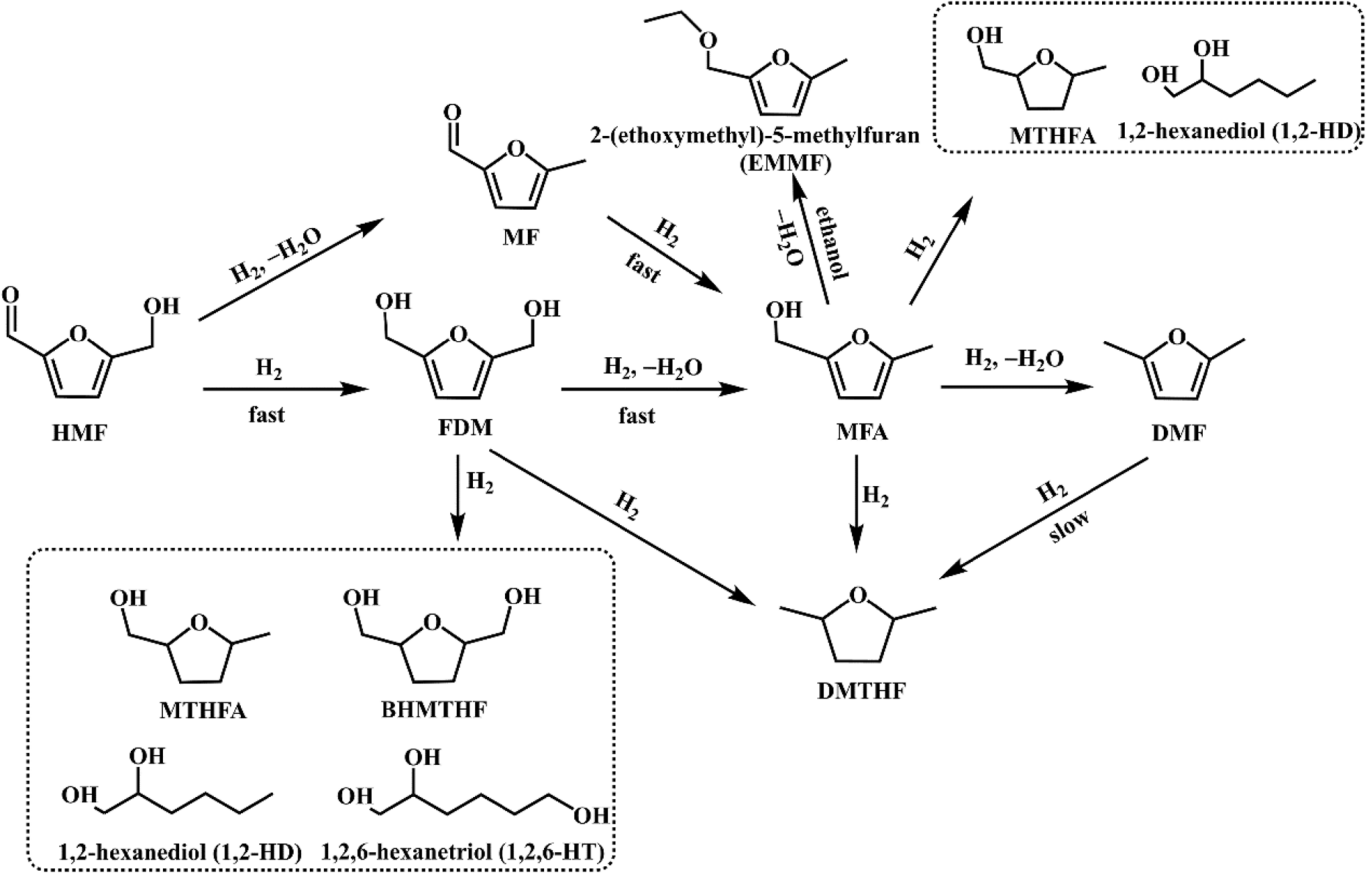
Possible reaction pathways for the hydrogenation of HMF in ethanol over copper doped porous metal oxide catalysts. Reproduced with permission from Ref. (Kumalaputri et al. 2014), copyright Wiley

**Scheme 3.**
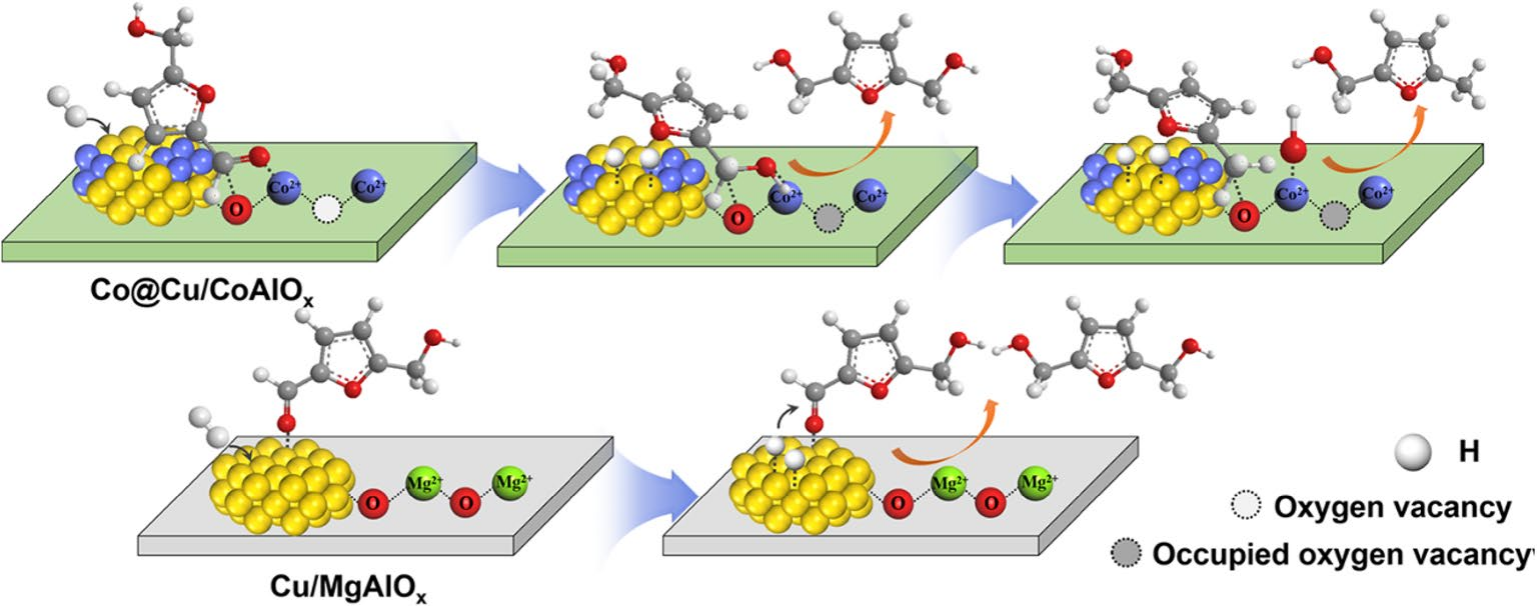
Possible Mechanism of HMF Hydrogenation over Cu/MgAlO_x_ and Co@Cu/CoAlO_x_ Catalysts. Reproduced with permission from Ref. (Wang et al. 2020). Copyright (2020) American Chemical Society

Compared to cobalt, copper is more abundant on earth. Copper catalysts have been extensively studied. Kumalaputri et al. (Kumalaputri et al. 2014) investigated the tunable and selective hydrogenation of HMF to different products over Cu-based catalysts. The introduction of Cu^2+^ during the preparation of hydrotalcite gave Cu-containing hydrotalcite, and the further calcination produced Cu-doped porous metal oxides. At 100 °C and 50 bar H_2_, a 99% selectivity of FDM and full conversion of HMF were obtained over Cu_0.59_Mg_2.34_Al_1.00_ in ethanol. The increase of temperature to 140 °C led to the decrease of FDM selectivity because of over-hydrogenation and other side-reactions. The addition of ruthenium dopant into the catalysts boosted the hydrogenation activity, which increased the total yield of DMF and DMTHF to 81%. Based on the identified reaction intermediates by GC–MS analysis and authentic standards, the authors proposed the reaction pathways for the conversion of HMF in ethanol in the presence of H_2_, as shown in Scheme 2. In addition to the hydrogenation of aldehyde group, hydroxyl group, and furan ring, etherification and ring-opening reactions also occurred. The reaction pathway and selectivity for HMF reduction over Cu-doped metal oxides could be tuned by adjusting interfacial structure, which was reported by Wang et al. (Wang et al. 2020). They prepared a series of catalysts with multiple interfaces by reduction of Cu-containing layered double hydroxides (LDHs). For example, Cu/MgAlO_x_ was derived from CuMg_5_Al_2_–LDHs. The Cu particles of Cu/MgAlO_x_ catalysts were partially encapsulated by the MgAlO_x_ support, resulting in the formation of highly intimate Cu–MgAlO_x_ interfaces. The specific Cu–MgAlO_x_ interfaces selectively activated C=O bond to produce FDM, affording a 92.7% yield. In contrast to the Cu/MgAlO_x_ catalyst, the Co@Cu/CoAlO_x_ catalyst with both Cu–MgAlO_x_ interfaces and metallic Co–Cu interfaces was prepared from CuCo_x_Al_2_–LDHs. By tuning the ratio of Co/Cu, the metallic Co–Cu interfaces could activate C–OH and even furan ring, offering 98.5% DMF and 83.6% DMTHF under optimized conditions. As shown in Scheme 3, the Cu particles of Cu/MgAlO_x_ adsorbed the carbonyl oxygen atom of HMF to form an on-top (O)–Cu linkage, which produces FDM as the sole product. The unsaturated O–Co–V_O_ (V_O_ represents an oxygen vacancy) sites of Co@Cu/CoAlO_x_ could interact with C=O or C–OH to form (C)–O–Co–(O) linkage, affording DMF as the product.

Some simple Cu-containing alloys also exhibited good catalytic performance. Bottari et al. (Bottari et al. 2015) found the commercial copper–zinc alloy nanoparticles with small size of < 150 nm could catalyze the selective hydrogenation of HMF to FDM in 95% yield in ethanol employing 70 bar H_2_. The direct use of commercial catalysts boosted the reproducibility of results regarding batch to batch variations. Almost at the same time, Zhu et al. (Zhu et al. 2015) reported the catalytic hydrogenation of HMF over homemade mineral-derived Cu–ZnO catalysts, affording 99.1% and 91.8% yields of FDM and DMF at 100 and 220 °C, respectively. At high temperature, such as 220  ^o^C, the deposition of organic compounds on the catalysts led to the deactivation, and the removal of undesirable deposited carbonaceous species could be achieved by calcination and reduction.

Cu species exhibited good catalytic performance for the reduction of HMF. In addition, some useful methods have been used for the preparation of Cu-based catalysts. Here, we introduced several typical examples. Rao et al. (Rao et al. 2021) developed a solvent-free solid-state grinding method to prepare copper–alumina catalysts for the selective hydrogenation of HMF, which avoids the tedious preparation procedure and the use of any solvents. The synergistic effect of Cu^0^ and Cu^2+^ as well as uniform distribution of copper NPs contributed to good catalytic performance, providing a 93% yield of FDM at 130 °C for 1 h in the presence of 30 bar H_2_. Another strategy for the preparation of Cu-based catalysts is to use metal organic frameworks (MOFs) as one of precursors. MOFs have some important natures, such as regular porosity, tunable pore size, the ability to stabilize metal/metal oxide NPs and prevent the agglomeration/growth of NPs (Falcaro et al. 2016), thereby becoming good alternatives as templates and/or precursors to fabricate heterogeneous catalysts. Feng et al. (Feng et al. 2019) reported ZIF-8 encapsulated Cu NP (CuNP@ZIF-8) catalysts for the reduction of HMF to FDM, affording a 99% yield in ethanol. Inductively coupled plasma optic emission spectrometer analysis and recyclability test suggested the good stability of highly dispersed Cu NPs which were immobilized by the frameworks of ZIF-8. Unlike the other studies (Rao et al. 2021), Feng et al. (Feng et al. 2019) demonstrated Cu^1^ and Cu^0^ species were responsible for the activation of aldehyde group and H_2_, respectively. The ligand is essential to the preparation of MOFs, and a large number of ligands are derived from non-renewable fossil resources. Bao et al. (Bao et al. 2022) employed renewable HMF-derived 2,5-furandicarboxylic acid as ligand to synthesize a novel Cu-based MOFs, and Cu NPs supported on carbon nanosheets were fabricated by post-treatment of the MOFs. The as-prepared Cu catalysts exhibited good catalytic activity and stability for the hydrogenation of HMF to FDM, providing a 99.4% yield.

In addition to catalysts, the reaction medium also influences product selectivity. Water is not only a cheap and green solvent, but also an important solvent additive to tune the product distribution. Liu et al. (Liu et al. 2015) studied the effects of water on the hydrogenation of HMF in THF in the presence of H_2_ by reaction kinetics, in which Cu/γ-Al_2_O_3_ was used as catalyst. High yield of DMF could be obtained in pure THF. Notably, the addition of small amount of water in THF (THF/water, *w*/*w*, *95*/*5*) led to water coverage on active sites, which remarkably inhibited the further hydrogenolysis of FDM to other products (i.e., DMF) and enhanced the selectivity of FDM. The negative effect of water on the reduction rate of HMF provides an approach to adjust the selectivity of different products. Nevertheless, when the production of DMF was carried out on large scale, the water formation changed the solvent components and influenced the catalytic performance.

**Scheme 4.**
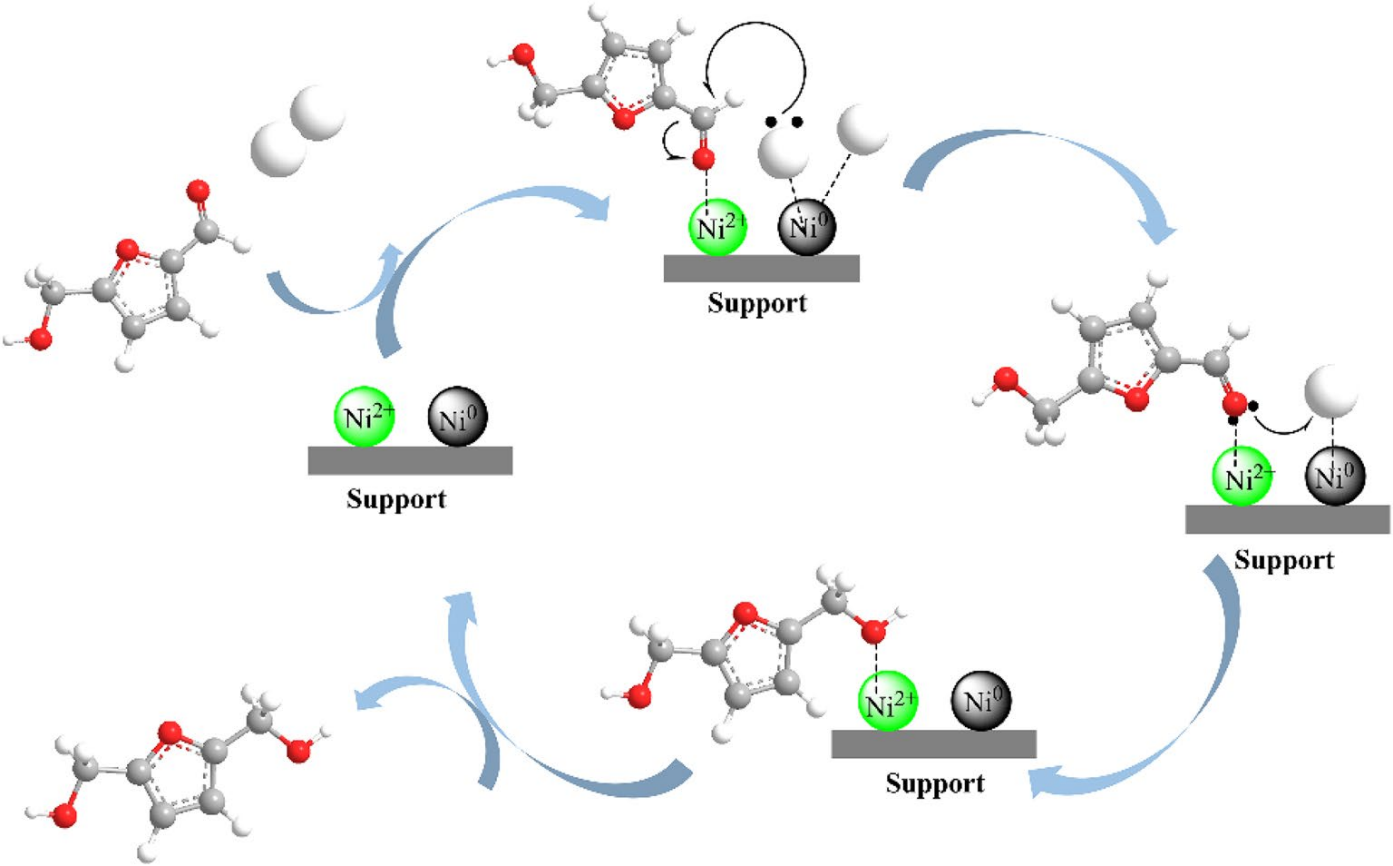
Possible mechanism of HMF hydrogenation over Ni/CNTs catalysts (Huang et al. 2022)

Ni-based catalysts have shown good catalytic performance in hydrogenation reaction. For the hydrogenation of HMF to FDM, several effective catalysts have been developed. Zhang et al. (Zhang et al. 2020) synthesized activated carbon (HC) employing sucrose as precursor by hydrothermal method, and the further loading of Ni component gave heterogeneous Ni/HC. The large surface area and a lot of oxygen-containing functional groups of the sucrose-derived carbon facilitated the dispersion and anchoring of Ni species. The metallic Ni species and big Ni NPs favored the selective hydrogenation of aldehyde group to yield FDM, and an 88% yield was obtained in 1,4-dioxane over reduced 10%-Ni/HC catalyst with big Ni particle size at 160 °C in the presence of 15 bar H_2_. The yield of FDM was acceptable, but the long reaction time of 24 h made it an energy-intensive process. Besides, the catalytic activity of the reused Ni/HC catalysts has not been reported, in spite of good thermal stability of the catalysts at reaction temperature. The use of carbon nanotubes (CNTs) as support of Ni afforded a 95% selectivity with 99.8% conversion in ethanol under milder conditions (5 bar H_2_, 120 °C, 3 h), which was reported by Liu’s group (Huang et al. 2022). In consistent with previous conclusion (Zhang et al. 2020), Ni^0^ could activate H_2_ and Ni^2+^ as Lewis acid could adsorb oxygen-containing groups, which synergistically catalyzed the formation of FDM, as shown in Scheme 4. However, the excessive Ni^2+^ species boosted deep reduction of HMF, decreasing the selectivity of FDM. The activity of spent Ni/CNTs catalysts decreased slightly due to the adsorbed organic compounds, and the re-calcination in 20% H_2_/Ar could partly recover the catalytic activity.

The impurities in substrate (i.e., HMF) might profoundly affect the catalytic performance. For example, DMSO has been widely used for the synthesis of HMF from carbohydrates because of its dissolving capacity and dehydration activity. Ojagh et al. (Ojagh et al. 2021) developed Ni/SiO_2_ catalysts with high Ni dispersion for the synthesis of FDM from HMF in excellent yield. The addition of DMSO led to remarkable decrease of both HMF conversion and FDM yield. The poisoning effect of DMSO could be explained by that the sulfur and carbon deposition covered the active Ni species. Owing to the great influence of DMSO, the choice of reaction medium for one-pot conversion of carbohydrates to downstream products of HMF should be considered seriously. Both the positive effect of DMSO for HMF synthesis and possible negative effect for HMF conversion should be taken into account.

Compared to monometallic catalysts, bimetallic catalysts have some potential advantages, such as tunable dispersion of NPs, adjustable hydrogenation activity, and facile isolation of catalysts. Several typical cases will be introduced as follow. Fe is a cheap metal and has been widely used to fabricate magnetic catalysis materials. Yu et al. (Yu et al. 2015) reported the hydrogenation of HMF over home-made bimetallic Ni–Fe/CNT catalysts. The introducing Fe species enhanced the dispersion of Ni component. In comparison with Ni/CNTs catalyst, an appropriate Ni/Fe ratio of 2.0 profoundly increased the selectivity of FDM from 76.4% to 96.1% at 110 °C for 18 h in 30 bar H_2_. The magnetic nature of Ni–Fe/CNT catalysts facilitated the separation of catalysts from reaction mixture for next catalytic run. Arias et al. (Arias et al. 2021) developed bimetallic CuFe NPs covered by thin carbon layers (denoted as CuFe@C) for the hydrogenation of HMF. Compared to monometallic Cu@C catalyst, the CuFe@C with an appropriate Cu/Fe ratio exhibited higher catalytic activity, which might be attributed to more active centers derived from the intimate contact between Cu and Fe.

Some typical examples of selective synthesis of FDM from HMF over non-noble metal catalysts using H_2_ as donor are summarized in Table 3.

**Table 3:** Some examples of catalytic hydrogenation of HMF to FDM over non-noble metal catalysts in the presence of H_2_

Catalyst	H_2_ (bar)	Solvent	T (^o^C)	t (*h*)	Conversion of HMF (%)	FDM yield (mol %)	Refs.
Co@C	10	Methanol	110	2	97	96	(Arias et al. 2020)
Co–NPs@SiO_2_	15	Methanol	70	16	–	95	(Chandrashekhar et al. 2022)
Co–NC	50	Solvent-free	90	3	99.3	91.5	(Wang et al. 2022c)
Cu_0.59_Mg_2.34_Al_1.00_	50	Ethanol	100	3	100	99	(Kumalaputri et al. 2014)
Cu/MgAlOx	10	1,4-Dioxane	180	5	97.3	92.7	(Wang et al. 2020)
CuZn	70	Ethanol	120	3	> 99	95	(Bottari et al. 2015)
Cu–ZnO	15	1,4-Dioxane	100	2	100	99.1	(Zhu et al. 2015)
20 mol% Cu–Al_2_O_3_	30	Methanol	130	1	> 99	93	(Rao et al. 2021)
CuNPs@ZIF-8	20	Ethanol	140	3	> 99	99	(Feng et al. 2019)
CFP-300	20	Ethanol	120	4	100	99.4	(Bao et al. 2022)
10%–Ni/HC	15	1,4-Dioxane	160	24	93.6	88	(Zhang et al. 2020)
15 wt % Ni/CNTs	5	THF	120	3	99.8	94.8	(Huang et al. 2022)
10Ni/SiO_2_	40	n-Butanol	180	6	100	99	(Ojagh et al. 2021)
Ni_2_–Fe_1_/CNTs	30	n-Butanol	110	18	100	96.1	(Yu et al. 2015)
Cu_0.24_–Fe_0.76_@C	10	Methanol	110	4	94	93	(Arias et al. 2021)

#### Alcohols or carboxylic acids as reduction reagent

Catalytic transfer hydrogenation (CTH) reaction has been regarded as a promising method for upgrading biomass-based carbonyl compounds, typically furfural and HMF, to their corresponding alcohols. Alcohols (i.e., methanol, ethanol, isopropanol) and carboxylic acids (typically formic acid) are used as hydrogen donors. Some recent advances in CTH of HMF have been summarized in this section.

**Scheme 5.**
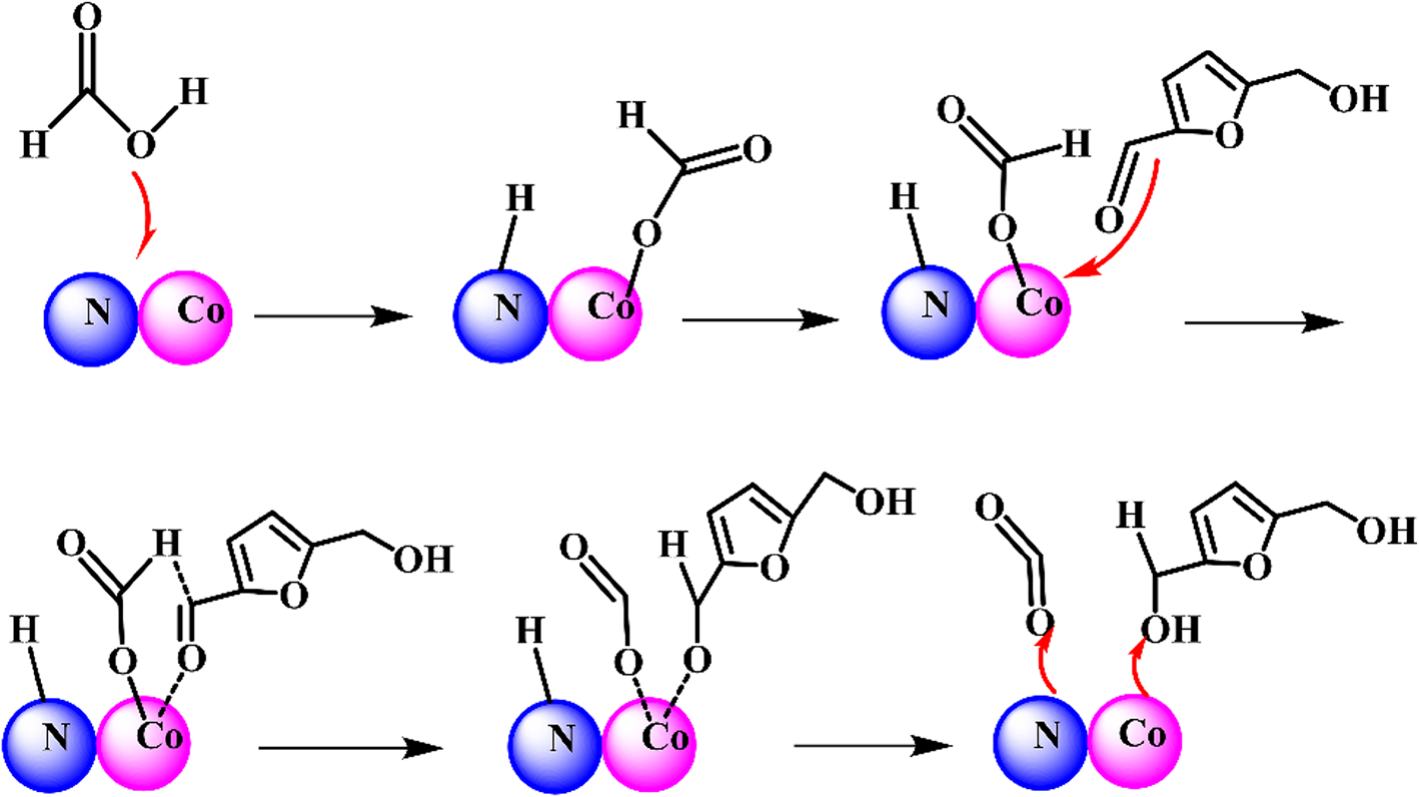
Proposed mechanism of CTH of HMF over Co–NC. Reproduced from Ref. (Xu et al. 2021), copyright Royal Society of Chemistry

Co-based catalysts are effective for CTH of HMF, in which either formic acid or isopropanol could act as hydrogen source. Xu et al. (Xu et al. 2021) developed N-doped carbon confined Co–N_x_ (Co–NC) catalysts employing SBA-15 as a mesopore template. A 92.3% selectivity of FDM with 88.2% HMF conversion was obtained in the presence of 7.5 equiv. formic acid at 160 °C for 5 h, while the conversion of formic acid reached to 89.5%. It is notable that highly dispersed N-confined Co species (Co–N_x_) instead of bare Co NPs acted as main active centers, as shown in Scheme 5. The CTH reaction starts from the dissociation of C–H in formic acid to form N–H* and Co-formate (Co–HCOO*) species, which is the rate-determining step. Then, the carbonyl oxygen of HMF is adsorbed by Co and interacts with the Co–HCOO* species. As a result, the transfer of hydrogen from formate to the carbonyl group proceeds via an intermolecular hydride transfer. Finally, the formation and desorption of FDM accompanies with the release of CO_2_. Wang et al. (Wang et al. 2022b) reported that Co_1.6_/UiO-66-NH_2_ catalyzed the effective CTH of HMF into FDM in isopropanol, affording a 95.9% FDM selectivity with 92.6% HMF conversion under milder reaction conditions (100 °C, 4 h). Among the investigated isopropanol, methanol, ethanol, n-butanol and 2-butanol, isopropanol provided the highest HMF conversion and FDM selectivity. The alcohols with low reduction potentials favored the CTH of HMF. Another important finding is that both acidic and basic sites of Co/UiO-66-NH_2_ catalysts have profound effect on catalytic activity, but the intrinsic mechanism is still unclear.

Hf-based catalysts have been reported for the CTH of HMF. Hu et al. (Hu et al. 2019) reported a new Hf-based metal–organic coordination polymer (Hf-DTMP) for the CTH of HMF to FDM in 2-butanol, providing a 96.8% yield at 130 °C for 4 h. The authors held a view that a large number of Lewis acid sites (Hf^4+^) and Lewis base sites (O^2–^) with synergistic effect contributed to the high catalytic activity. The results of control experiments revealed that Lewis base sites played more important roles.

Huang et al. (Huang et al. 2023) reported an example using CuO/boehmite for the CTH of HMF into FDM, in which ethanol was employed as both solvent and hydrogen donor. Boehmite contains both acid and base sites, which has a significant effect on catalytic performance. For instance, 2-hydroxymethyl-5-ethoxyfuran (HEMF) might be produced as a by-product from FDM and ethanol catalyzed by the hydroxyl group Brønsted acid (H^+^) on boehmite surface. When boehmite was used as a sole catalyst at 160 °C, the selectivities of FDM and HEMF were 59.7% and 25.1%, respectively. The addition of CuO hindered the Brønsted acid sites of boehmite and introduced Lewis acid sites (Cu^2+^) on the catalyst surface. The increase of the Lewis acid/Brønsted acid ratio inhibited the etherification of alcohols and boosted the selectivity of FDM. As a result, a 96.9% selectivity of FDM with 75.9% conversion was obtained over 40 wt% CuO/boehmite under the same conditions. In combination with other findings (Wang et al. 2022b; Hu et al. 2019), more efforts should be devoted to tuning acid–base properties of catalysts for the enhancement of FDM formation. The reduction of CuO/boehmite by ethanol led to the decrease of catalytic activity, and the re-calcination in air recovered its activity.

**Scheme 6.**
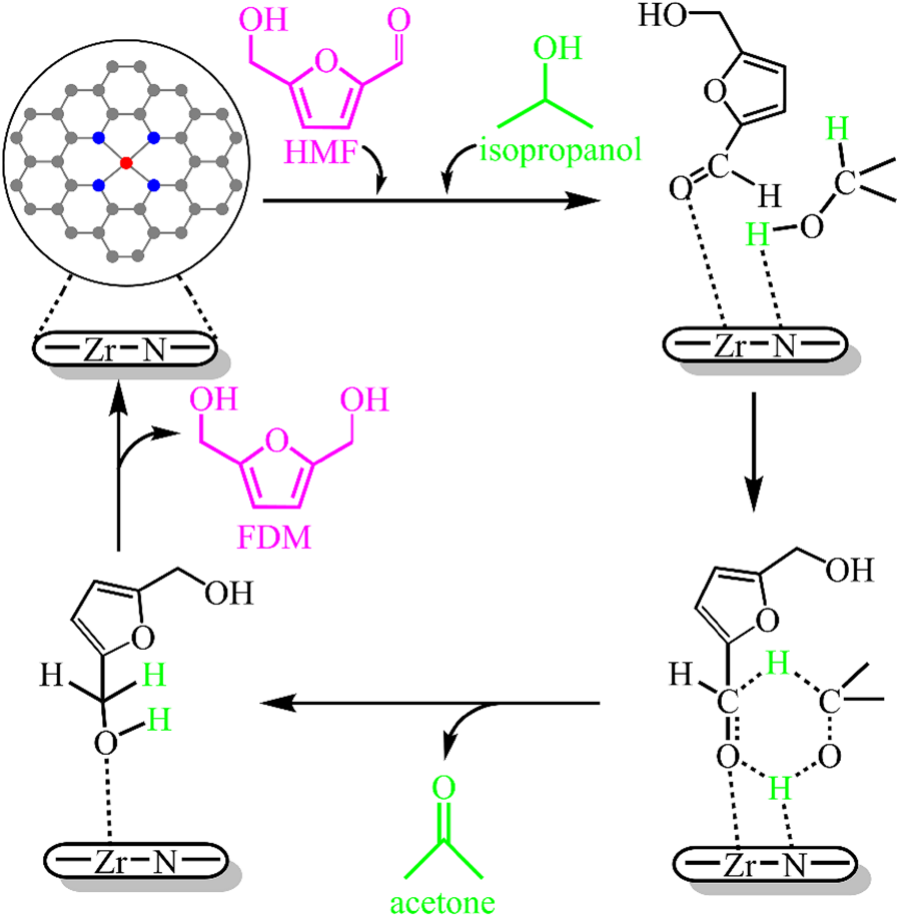
Reaction mechanism for the CTH of HMF to FDM in isopropanol over Zr/NC. Reprinted from Ref. (Hu et al. 2022), copyright Royal Society of Chemistry

The use of single atom catalysts for biomass valorization has attracted attention (Mondelli et al. 2020). For instance, Hu et al. (Hu et al. 2022) prepared Zr-based single-atom catalyst (Zr/NC) with low Zr content of 0.58 wt% by pyrolysis of Zr-doped ZIF-8. The Zr/NC catalyst could catalyze the CTH of HMF to FDM, offering an approaching 100% selectivity with full conversion at 130 °C for 2.5 h in isopropanol. The use of ethanol or n-butanol also afforded satisfactory yields of FDM up to 95%. The atomically dispersed Zr–N_4_ species were confirmed to be the active sites, as shown in Scheme 6. Both the electron transfer from the pyridinic-N to Zr in the Zr–N_4_ active sites and high Lewis acid–base strengths of Zr/NC contributed to the good catalytic activity. The Zr–N_4_ structures were stable under investigated conditions, but the deposition of carbon on the spent Zr/NC led to gradual decrease of FDM yield.

**Scheme 7.**
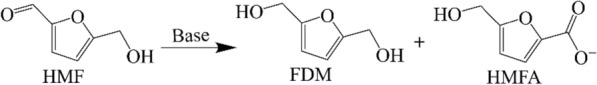
Synthesis of FDM from HMF by Cannizzaro reaction

**Scheme 8.**
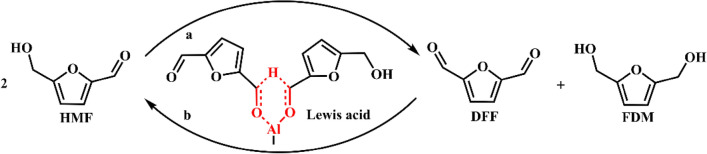
Lewis acid-catalyzed MPVO reaction of HMF to DFF and FDM. Reproduced with permission from Ref. (Li et al. 2017), copyright Wiley

As discussed earlier, alcohols can act as a hydrogen donor for the reduction of HMF to FDM. A special example is Cannizzaro reaction. HMF contains an aldehyde group without a hydrogen atom at an α-position. The base-induced Cannizzaro reaction of HMF generates FDM and 5-hydroxymethylfuranoic acid (HMFA), as illustrated in Scheme 7. Some typical strong bases, such as NaOH, KOH, Ca(OH)_2_, have been used and provided good yields (Subbiah et al. 2013; Kang et al. 2012). For example, Subbiah et al. (Subbiah et al. 2013) investigated the conversion of HMF in water in the presence of 1.1 equiv. NaOH at room temperature, and an 86% yield of FDM as well as 87% HMFA was obtained after 18 h. The generated FDM and HMFA could be isolated by selective crystallization. Similar to Cannizzaro reaction, the selective conversion of HMF to 2,5-diformylfuran (DFF) and FDM is another strategy. Li et al. (Li et al. 2017) investigated a special Meerwein–Ponndorf–Verley–Oppenauer (MPVO) reaction to produce DFF and FDM catalyzed by Al-based Lewis acid, where HMF acted as both oxidant and reductant. In such a process, a reversible equilibrium exists between HMF and DFF/DHMF, as illustrated in Scheme 8, resulting in low conversion. Owing to the lower solubility of FDM compared to DFF in acetonitrile, the use of acetonitrile as reaction medium facilitated the crude separation of FDM products from DFF products, thereby pushing the equilibrium shift towards HMF conversion. Under optimized reaction conditions, a 44.7% conversion of HMF with DFF/FDM molar ratio as 1.0 could be obtained. The use of HMF as hydrogen donor avoids external reductant, but the co-existence of oxidized product and FDM makes it more difficult to purify the products.

Some examples of CTH of HMF to FDM are summarized in Table 4.

**Table 4:** Some examples of CTH of HMF to FDM

Catalyst	H source	Solvent	T (^o^C)	t (h)	Conversion of HMF (%)	FDM yield (mol%)	Refs.
Co–NC	Formic acid	1,4-Dioxane	160	5	88.2	81.4	(Xu et al. 2021)
Co–NC	Formic acid	1,4-Dioxane	160	10	100	86	(Xu et al. 2021)
Co_1.6_/UiO-66-NH_2_	Isopropanol	Isopropanol	100	4	92.6	88.8	(Wang et al. 2022b)
Hf-DTMP	2-Butanol	2-Butanol	130	4	99.1	96.8	(Hu et al. 2019)
40 wt% CuO/Bhm	Ethanol	Ethanol	160	3	75.9	73.5	(Huang et al. 2023)
Zr/NC	Isopropanol	Isopropanol	130	2.5	100	99.6	(Hu et al. 2022)

#### Silane as reduction reagent

**Scheme 9.**
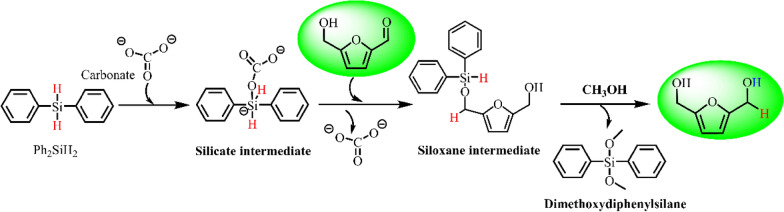
Plausible reaction pathway for the HMF–FDM conversion. Reprinted from Ref. (Long et al. 2018), copyright MDPI

The silanes are water- and air-stable hydrogen sources and could be stimulated by metal-containing catalysts under mild conditions. Long et al. (Long et al. 2018) studied the selective reduction of HMF to FDM over alkali metal salts and alkaline earth metal salts using diphenylsilane as hydrogen source at room temperature. Among screened catalysts, K_2_CO_3_ offered the full conversion of HMF with a 70.1% yield of FDM after 2 h. The relatively low yield of FDM could be attributed to the formation of stable siloxane intermediate. The addition of methanol facilitated the breakage of the siloxane bond to form FDM, boosting the yield of FDM to 94.2%. A possible reaction pathway has been shown in Scheme 9. The reduction reaction starts from the interaction of CO_3_^2–^ and diphenylsilane to form a silicate intermediate with nucleophilicity. Then, the silicate intermediate activates the aldehyde group of HMF through hydride transfer to afford the siloxane intermediate, and the further hydrolysis generates FDM.

### Synthesis of FDM from HMF by photo-catalysis

**Scheme 10.**
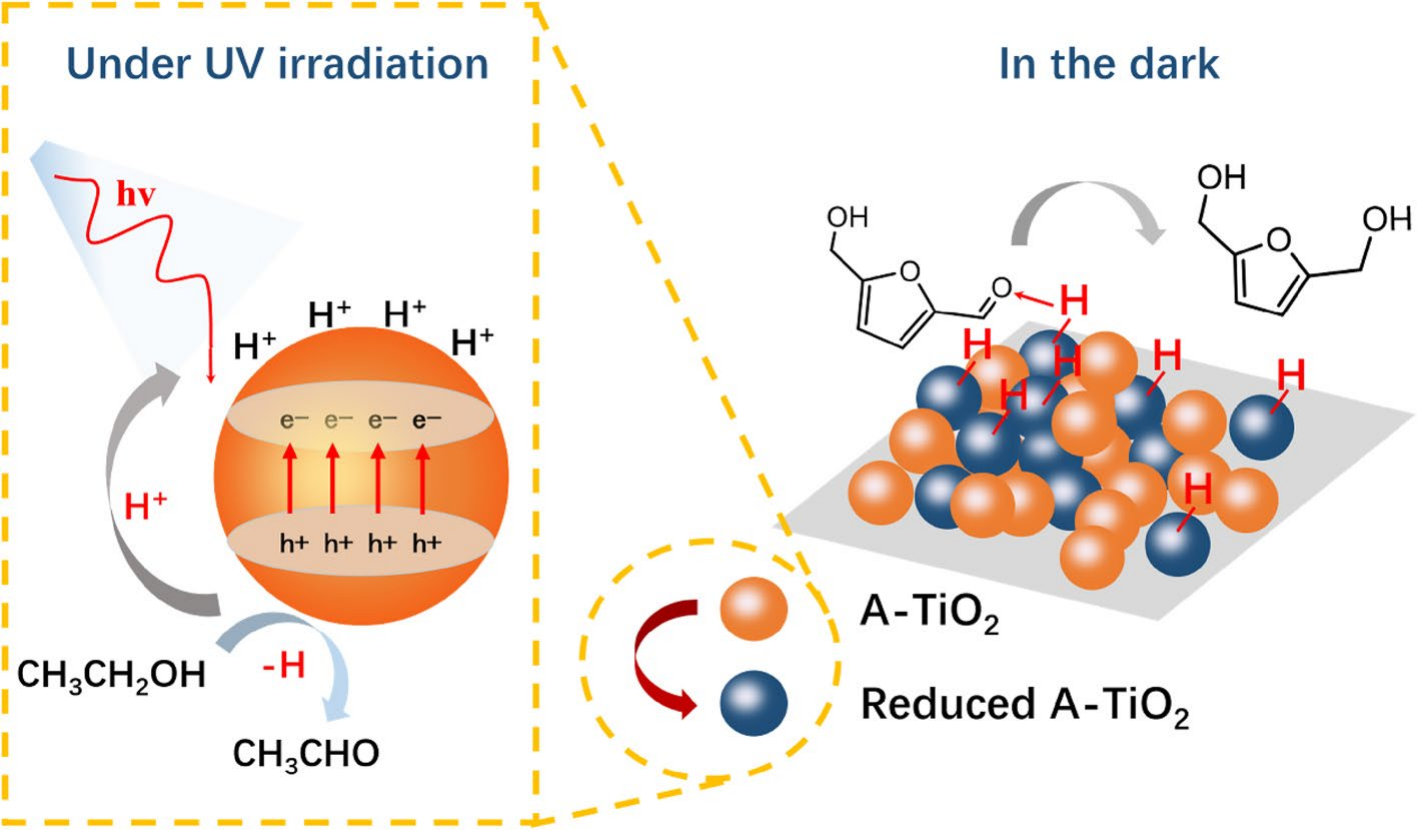
Possible mechanism of HMF hydrogenation to FDM over amorphous TiO_2_. Reprinted from Ref. (Qiao et al. 2019), copyright Royal Society of Chemistry

Photo-catalysis provides a green and sustainable approach for the production of a variety of valuable compounds from biomass. Some cases about photo-catalytic reduction of HMF have been reported. Guo et al. (Guo and Chen 2016) reported the photo-induced reduction of HMF over a Pt/g-C_3_N_4_ catalyst under visible light irradiation. Under optimized conditions, only a 6.5% yield of FDM was obtained. This is an early example employing photo-catalysis for the synthesis of FDM, and the catalytic efficiency was still unsatisfactory. Photo-induced holes and/or reactive oxygen species are sensitive to reaction conditions, which might induce undesirable side reactions. Qiao et al. (Qiao et al. 2019) proposed a two-step strategy for the synthesis of FDM from HMF on amorphous TiO_2_ using ethanol as hydrogen donor, as shown in Scheme 10. The first step was carried out to store H^+^/e^−^ species on the surface of TiO_2_ under UV irradiation, and acetaldehyde was formed by dehydrogenation of ethanol. Then, the second step was triggered in the dark after the addition of HMF. The oriented adsorption of the HMF aldehyde group and the enhanced desorption of FDM by acetaldehyde inhibited undesirable deep reduction, which increased the selectivity of FDM. A 99% conversion of HMF with a 99% selectivity of FDM was obtained under N_2_ atmosphere at 25 °C. Jaryal et al. (Jaryal et al. 2022) reported the photo-catalytic reduction of HMF over TiO_2_ in acetonitrile at room temperature using p-methoxybenzyl alcohol as hydrogen donor, and a 30% yield of FDM was achieved simultaneously with the formation of 4-anisaldehyde after 24 h.

### Synthesis of FDM from HMF by electro-catalysis

If the cheap electricity is obtained from green and sustainable resources, such as solar and wind, electro-catalysis will be a potential future green methodology for biomass utilization. Especially when water acts as a proton source, electro-catalysis is regarded as a promising hydrogenation strategy. Some solid mono-metal electrodes have been used as catalysts. Koper’s group (Kwon et al. 2013) investigated the hydrogenation of HMF over solid metal electrodes in neutral media. Among the studied metals, Ag electrode favored the selective production of FDM with selectivity up to 85% at all potentials. The highest concentration of FDM on Ag reached 13.1 mM cm^−2^ at − 0.81 V. Apart from electrodes, the applied potential also affected the product selectivity. The more negative potentials boosted the evolution of more hydrogen, thereby decreasing the selectivity of FDM. The pH value played an important role in terms of onset potential and final product. The same group (Kwon et al. 2015) demonstrated the electro-catalytic hydrogenation of HMF in 0.5 M H_2_SO_4_ aqueous solution, and the further hydrogenation of furan ring was remarkably promoted because of lower activation energy under acidic conditions compared to neutral conditions.

The combination of Ag and an additional metal or metal oxide was used to enhance the hydrogenation of HMF (de Luna et al. 2020, 2021; Zhong et al. 2022; Zhao et al. 2022). For example, Zhong et al. (Zhong et al. 2022) developed Ag-decorated Cu nanowire arrays as electro-catalysts. A 97.1% FDM selectivity with 99.5% HMF conversion and 96.5% faradaic efficiency was achieved. The synergic role of Cu and Ag active sites promoted the rapid ion diffusion, which enhanced the electro-catalytic activity for selective synthesis of FDM. Zhao et al. (Zhao et al. 2022) reported dispersed Ag nanoparticles immobilized on one-dimensional TiO_2_ nanotubes for the electro-reduction of HMF, affording an 89.8% selectivity of FDM as well as a productivity value of 0.1 mmol cm^−2^ h^−1^. The heterojunction-induced interactions between Ag and TiO_2_ disfavored the hydrogen evolution reaction and facilitated electron transfer across the interface. Moreover, the use of TiO_2_ as a support increased the utilization efficiency of Ag. These beneficial factors enhanced the catalytic performance.

**Scheme 11.**
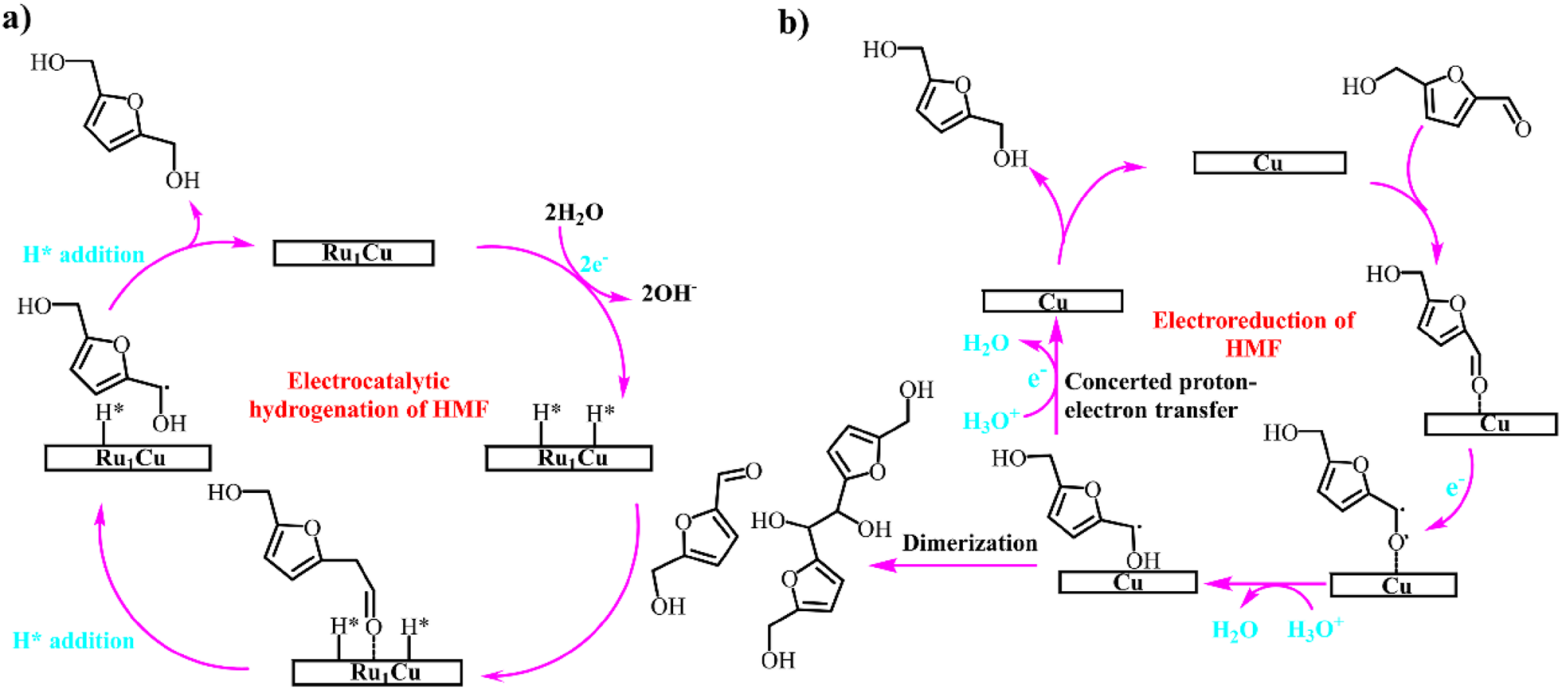
Proposed mechanisms of HMF hydrogenation to FDM over a)Ru_1_Cu and b)Cu. Reproduced with permission from Ref. (Ji et al. 2022), copyright Wiley

The effective activation of water to form active H species and the inhibition of hydrogen evolution are of significance for selective synthesis of FDM. Ji et al. (Ji et al. 2022) reported that Ru_1_Cu single-atom alloy catalyst showcased better performance for the reduction of HMF to FDM with boosting productivity (0.47 vs. 0.08 mmol cm^−2^ h^−1^) and faradic efficiency (85.6 vs. 71.3%) compared to Cu counterpart. It is notable that a high faradic efficiency of 87.5% could be obtained at high HMF concentration (100 mM). As shown in Scheme 11, the additional dispersed Ru switches the reaction pathways. The single-atom Ru enhanced the activation of water to generate H* species that rapidly react with HMF to form FDM. In contrast, the carbonyl of HMF accepted an electron to form a radical intermediate that could be further converted into FDM or dimer.

## Synthesis of FDM from hexose

**Scheme 12.**
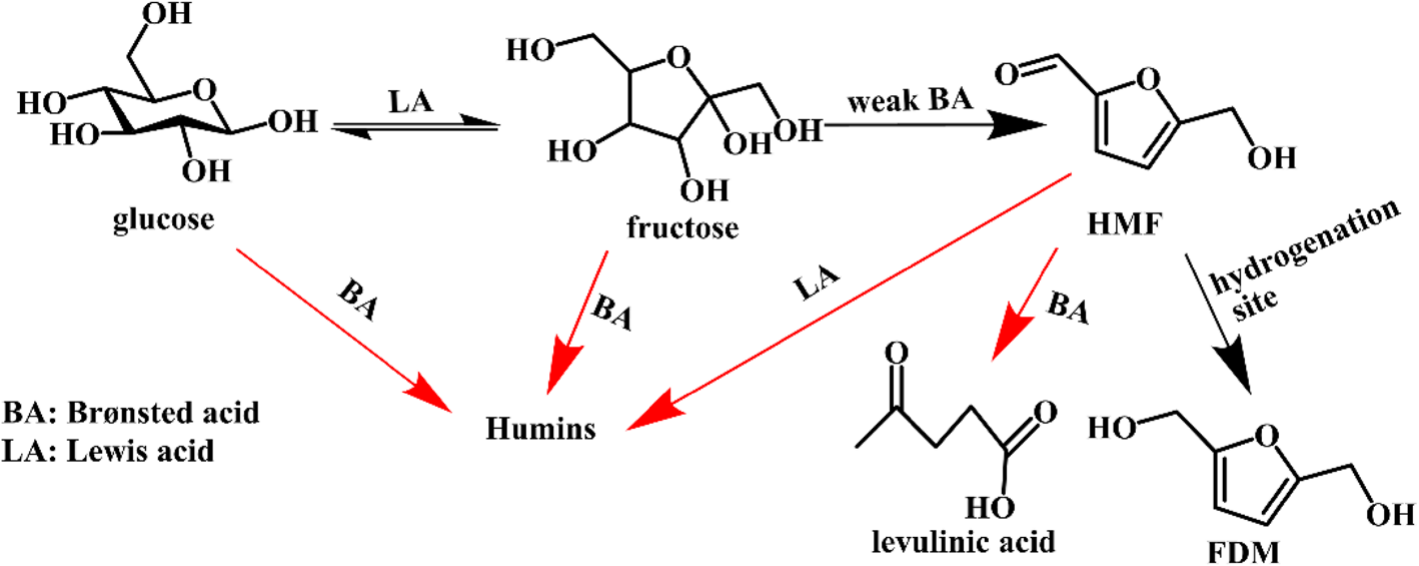
Possible reaction network of FDM synthesis from hexose

The selective hydrogenation of HMF has been widely studied, and high yields of FDM have been obtained in some cases. However, HMF is still not an accessible bulk chemical because of difficult purification process and the unstable nature. The direct conversion of carbohydrates to FDM without separation of HMF intermediate is an alternative method, but it faces challenges. It is well-known that HMF is usually produced by acid-catalyzed dehydration of hexose (typically fructose and glucose). Many undesirable side-reactions occur in the presence of acids, resulting in the formation of by-products, such as levulinic acid, formic acid, lactic acid, and humins, as shown in Scheme 12. Moreover, the direct hydrogenation of carbohydrates to corresponding sugar alcohols is also a possible side-reaction. Limited works on this topic have been reported. In addition, some typical examples will be introduced next.

**Scheme 13.**
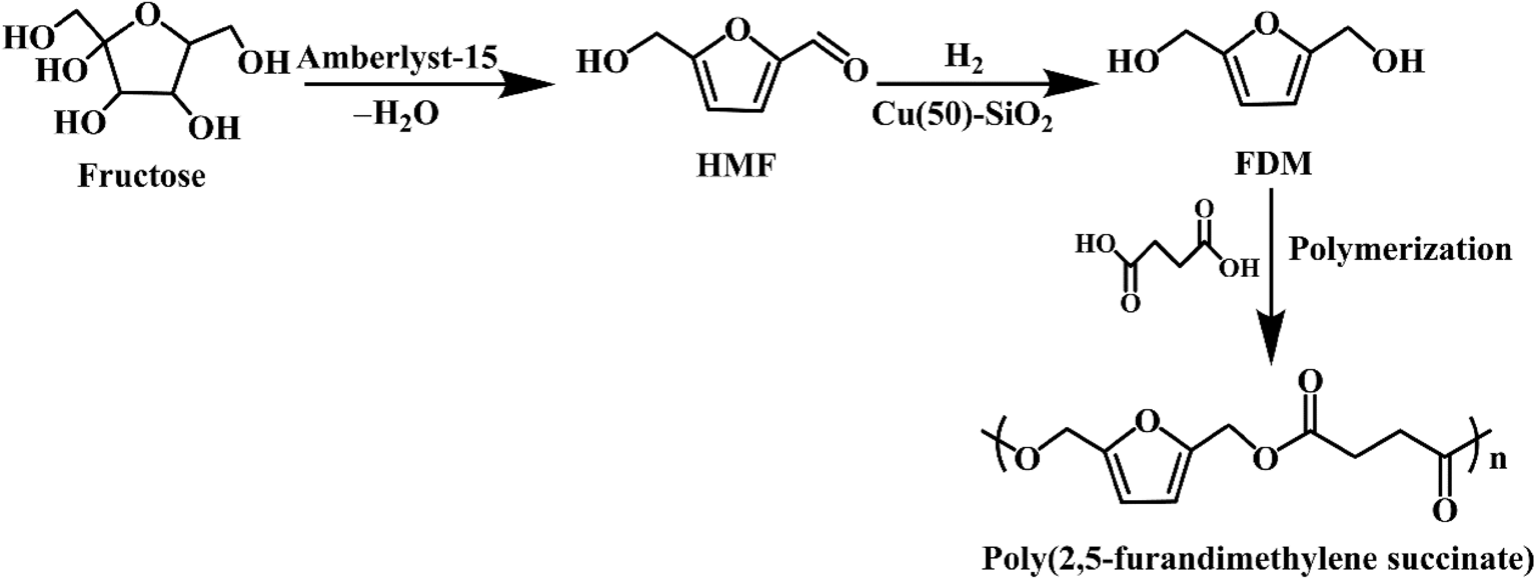
Conversion of fructose to Poly(2,5-furandimethylene succinate) without purification of FDM (Upare et al. 2018)

Fructose is a widely used sugar for the synthesis of HMF. Some efforts have been devoted to the production of FDM from fructose. Owing to different requirements of fructose dehydration to HMF and HMF hydrogenation to FDM, in some examples, these two steps are carried out under different conditions, respectively. The biomass-derived formic acid can act as an acid catalyst for fructose dehydration and a hydrogen donor for hydrogenation. In 2010, Thananatthanachon et al. (Thananatthanachon and Rauchfuss 2010) reported a two-step method for the synthesis of FDM from fructose. Formic acid catalyzed the dehydration of fructose to HMF in DMSO at 150 °C for 5 h. Then, the hydrogenation of as-formed HMF was carried out at 40 °C after adding excess formic acid (in THF), triethylamine, and Ir catalysts, providing an isolated 71% yield of FDM. Cai et al. (Cai et al. 2014) demonstrated one-pot tandem reactions in ionic liquid/water system. The dehydration of fructose was carried out in 1-butyl-3-methylimdazolium chloride ([BMIm]Cl) at 130 °C for 20 min, providing a 75% yield of HMF. Then, the hydrogenation of resulting HMF was performed after adding water and supported metal catalysts. The selectivity of products was strongly dependent on the the metal species. Under 60 bar H_2_ at 50 °C for 3 h, the use of 5% Ir/C and 5% Pd/C afforded 70% and 84% selectivities of FDM (based on HMF) and BHMTHF, respectively. Upare et al. (Upare et al. 2018) reported an integrated protocol for the synthesis of FDM-based polymer from fructose. FDM was produced from fructose by sequential dehydration and hydrogenation in n-butanol, in which heterogeneous Amberlyst-15 and Cu–SiO_2_ were used, respectively. A 92% yield of FDM based on fructose was obtained. Moreover, the mixture of FDM/n-butanol was directly converted into poly(2,5-furandimethylene succinate) employing biomass-derived succinic acid as another monomer without purification of FDM, as illustrated in Scheme 13. This work provided an successful example for the production of the down-stream product of FDM from fructose, and no nobel metal catalysts were used.

Compare to “one-pot, two step” method, “one-pot, one step” processes usually afford lower yield of FDM. Xiang et al. (Xiang et al. 2016) reported that the combination of HY zeolite and hydrotalcite (HT)–Cu/ZnO/Al_2_O_3_ catalyzed the one-step conversion of fructose towards FDM in a fixed-bed reactor. H_2_ was used as the hydrogen donor. At low temperature, HT–Cu/ZnO/Al_2_O_3_ catalyst selectively adsorbed the aldehyde group of HMF, affording FDM as the dominant reduction product. The increase of temperature promoted the hydrogenolysis of hydroxyl group, resulting in DMF as the dominant reduction product. Under optimized conditions, a 48.2% yield of FDM and a 40.6% yield of DMF were obtained at 140 °C and 240 °C, respectively. However, the moderate HMF yield in dehydration step limited the overall yield of FDM. Xu et al. (Xu et al. 2022b) used formic acid as acid catalysts for the dehydration of fructose to HMF and hydrogen donor for hydrogenation, while heterogeneous Co ctalysts supported on nitrogen-containing carbon were used as hydrogenation catalyst. Under optimized conditions (170 °C, 3 h), an enhanced FDM yield of 60.2% with full conversion was achieved. Compared to sole water or 1,4-dioxane as solvent, the use of water/1,4-dioxane medium favored the formation of FDM from fructose by facilitating the solubility of fructose and inhibiting the undesirable condensation or polymerization of HMF or FDM. The dehydration of fructose to HMF was the rate-determining step of the cascade conversions, which has been revealed by kinetic studies. The same group (Xu et al. 2022a) reported the selective conversion of glucose to FDM using NiCl_2_ as dehydration catalyst in a similar catalytic system, and a 60% FDM yield was afforded at 160 °C for 6 h. Moreover, moderate FDM yields of 46.4–56.1% could be obtained from di/polysaccharides, including sucrose, lactose, maltose, glucan, and cellobiose.

Some typical examples for direct conversion of carbohydrates to FDM are summarized in Table 5.

**Table 5:** Some examples of one-pot production of FDM from hexose

Hexose	Reaction conditions	Yield (mol%, based on monosaccharide)	Refs.
Fructose	The 1st step: 20 mmol fructose, 1 mL DMSO + 10 mL THF, 2 mol formic acid, 150 °C, 5 h The 2nd step: 2 mmol triethylamine, 0.1 mmol iridium catalyst, 40 C, 5 min, stirred. Then, a solution of formic acid (0.88 mL, 20 mmol) in THF (20 mL) was added using a syringe pump (flow rate = 2.5 mL h^–1^)	71 (isolated)	(Thananatthanachon and Rauchfuss 2010)
Fructose	The 1st step: 1 mmol fructose, 0.5 g [BMIm]Cl, 130 °C, 20 min The 2nd step: 35 mL cool water, 50 mg 5% Ir/TiO_2_, 60 bar H_2_, 50 °C, 3 h	71	(Cai et al. 2014)
Fructose	The 1st step: 15 g fructose, 1.0 g Amberlyst-15, 85 g n-butanol, 100 °C, 5 h The 2nd step: 50 g HMF feed from the fructose dehydration, 0.5 g reduced Cu(50)–SiO_2_, 15 bar H_2_, 100 °C, 5 h	92	(Upare et al. 2018)
Fructose	WHSV (fructose) = 0.02 h^–1^, H_2_ = 15 mL/min, 1 bar, 4 g HY zeolite and 5 g CuO/ZnO/Al_2_O_3_, 140 °C; fructose/water/γ-butyrolactone, *w*/*w*/*w*, *3*/*15*/*82*	48.2	(Xiang et al. 2016)
Fructose	30 mg fructose, 20 mg Co–NC, n_formic acid_/n_fructose_ = 15, 3.5 mL water–1,4-dioxane solvent (3/7), 5 bar N_2_, 170 °C, 3 h, 500 rpm	62	(Xu et al. 2022b)
Glucose	30 mg glucose, 20 mg Co–NC, 5 mg NiCl_2_, n_formic acid_/n_fructose_ = 20, 3.5 mL water-1,4-dioxane solvent (3/7), 5 bar N_2_, 160 °C, 6 h, 500 rpm	60	(Xu et al. 2022a)

## Conclusions and perspectives

### Conclusions

FDM is an attractive biomass-derived chemical that can be applied in many fields. The synthesis of FDM from HMF, an important platform compound, has been extensively investigated. Both noble and non-noble metal catalysts have showcased good catalytic activity for the hydrogenation of HMF towards FDM in satisfactory yields in some cases. In such a hydrogenation transformation, H_2_ is usually used as a hydrogen source. Apart from H_2_, formic acid and some alcohols also could act as a hydrogen donor, which avoids the use of high pressure H_2_ and makes the reduction reaction safer. In addition to traditional thermo-catalysis, emerging technologies including photo-catalysis and electro-catalysis provide alternative approaches for the synthesis of FDM. The direct conversion of carbohydrates (typically fructose and glucose) is a much more promising strategy for the production of FDM, but only limited examples have been reported. Challenges still exist in improving FDM yield from carbohydrates.

### Perspectives

Although many interesting examples have been reported, the production of FDM on industrial scale still faces enormous challenges. Some typical issues should be addressed in the future studies. (1) Considering the high cost of HMF, future research should be focused on developing new effective catalytic systems for the direct transformation of carbohydrates to FDM. Currently, the single step either the dehydration of carbohydrates to HMF or the hydrogenation of HMF to FDM has been widely investigated, but the cascade conversion suffers from serious side-reactions. (2) To design an effective catalytic system for one pot synthesis of FDM should synthetically consider several factors, such as reaction medium, catalyst, gas atmosphere, reaction temperature, etc. How to combine these conditions is difficult but of significance. To develop effective bifunctional catalyst or combination catalysts is attractive, which might afford selective synthesis of FDM in a one-step protocol. For potential application in industry, the design of equipment also should be taken into account in accordance with respective practical situations. (3) The deep insight for the reaction mechanism of dehydration and hydrogenation steps by theoretical or experimental method will be helpful for rational design. The fundamental mechanism insights are essential to develop more efficient and stable catalysts. (4) The systematic establishment of separation technologies is important. In most cases, FDM need be separated from reaction mixture before further application. Unfortunately, previous studies mainly focused on high yield of target products. Therefore, to develop energy-efficient separation technologies is of significance. (5) In addition to boosting catalysis efficiency, the cost and environmental influence also should be considered for potential industrial applications.

## Data Availability

Not applicable.
